# Effects of pH on opioid receptor activation and implications for drug design

**DOI:** 10.1016/j.bpj.2024.07.007

**Published:** 2024-07-05

**Authors:** Christoph Stein

**Affiliations:** 1 Charité Universitätsmedizin Berlin, Campus Benjamin Franklin, Experimental Anaesthesiology, Berlin, Germany

## Abstract

G-protein-coupled receptors are integral membrane proteins that transduce chemical signals from the extracellular matrix into the cell. Traditional drug design has considered ligand-receptor interactions only under normal conditions. However, studies on opioids indicate that such interactions are very different in diseased tissues. In such microenvironments, protons play an important role in structural and functional alterations of both ligands and receptors. The pertinent literature strongly suggests that future drug design should take these aspects into account in order to reduce adverse side effects while preserving desired effects of novel compounds.

## Significance

Structure-based drug design has yielded novel opioid agonists that selectively activate receptors in injured, but not normal, tissues. These findings have important implications and may enable the development of improved GPCR drugs devoid of adverse side effects elicited in noninjured organs.

## Introduction

G-protein-coupled receptors (GPCRs) are integral membrane proteins that transduce chemical signals from the extracellular matrix into the cell. They are the targets of over one third of all drugs available today (1,2). A prominent GPCR subgroup is opioid receptors. Opioid receptors are expressed throughout the nervous system and in many other organs (e.g., endocrine, immune, and intestinal systems). These receptors can be targeted by a large variety of endogenous (peptide) and exogenous (plant-derived or synthetic) opioid ligands (2,3,4,5). For centuries, opioid agonists have been the most efficient drugs for the treatment of severe pain. Conventional opioids (e.g., morphine, fentanyl) produce pain relief (analgesia) by the activation of mu-opioid receptors (MORs; the clinically most important opioid receptor type) and consequent modulation of downstream signaling molecules (G proteins, membrane ion channels) in central and peripheral sensory neurons (3,4,5,6,7). Unfortunately, this is accompanied by serious adverse effects such as addiction, dependence, cardio-respiratory arrest, locomotor disturbance, sedation, nausea, and/or constipation. These side effects are mediated by opioid receptors in the brain or intestine. In addition, tolerance (i.e., diminishing effects with repeated administration of opioid ligands) to all desired and undesired actions (e.g., analgesia, respiratory depression, nausea, sedation) can develop (4,5,6,8).

In view of these detrimental effects and the opioid crisis resulting therefrom, extensive research efforts are ongoing to find ways of eliminating adverse actions while preserving analgesia. A particularly promising—and clinically proven—approach is the selective (exclusive) activation of opioid receptors on peripheral sensory neurons innervating injured tissue (i.e., the source of pain generation) while leaving out opioid receptors in healthy environments (e.g., brain, intestinal wall) (4,6,9,10,11,12,13,14,15).

Injured tissue (resulting from, e.g., surgery, trauma, arthritis, colitis, cancer, infection, or lack of oxygen delivery) is accompanied by inflammation. This diseased milieu is made up of increased amounts of various cells (e.g., neutrophils, lymphocytes, macrophages) and mediators (e.g., cytokines, chemokines, extravasated proteins, prostaglandins, cyclooxygenase, oxygen radicals, lactate, protons) and is characterized by acidosis (pH < 7.35) (16,17,18) (Table 1). Importantly, protons are not only capable of directly activating so-called “proton-sensing” GPCRs (19,20) but can also influence interactions between different GPCRs and their cognate ligands (21,22). The present article will focus on the latter. In summary, current evidence suggests that future drug design should depart from analyzing the structure and function of GPCRs only under normal conditions. More attention should be paid to differences between healthy and diseased microenvironments (e.g., hypoxia, ionic strength).

**Table 1:** pH values in injured/inflamed tissues measured in vivo/ex vivo

Species, tissue	Lowest pH	Reference
**Infectious agents**		

Human, abscess	5.4	(23)
Guinea pig, intraperitoneal bacterial inoculation	5.6	(24)
Mouse, subcutaneous/intraperitoneal bacterial inoculation	5.8	(24)
Rat, air pouch granuloma induced by carrageenan, dextran, staph. aureus	6.87	(25)
Human, abdominal abscess	6.0	(26)
Human, peritoneal fluid, abdominal infection	5.9	(27)
Rat, Freund’s adjuvant paw inflammation	6.8	(28)
Rat, paw incision	7.02	(28)
Rat, Freund’s adjuvant paw inflammation	6.82	(29)
Rat, Freund’s adjuvant paw inflammation	6.84	(30)

**Noninfectious agents**		

Dog, turpentine-induced pleural exudate	6.6	(31)
Dog, turpentine-induced pleural exudate	6.5	(32)
Dog, turpentine-induced pleural exudate	6.0	(33)
Rabbit, diabetic skin wounds	6.9	(34)
Rabbit, brain, wounds, ischemia	5.0	(35)
Rat, ischemic heart, intracellular	5.7	(36)
Rat, seminiferous tubules and epididymis	6.57 ± 0.08	(37)
Human, exercised muscle, intracellular pH	6.1	(38)
Human, exercised muscle, calculated intracellular pH	6.31 ± 0.09	(39)
Rat, carrageenan inflammation, aspirated	6.94	(40)
Human, exercised muscle, intracutaneous pH	6.67	(41)
Human, atherosclerotic plaque	7.15 ± 0.01	(42)
Rabbit, aorta	7.40 ± 0.43	(42)
Human, umbilical artery	7.24 ± 0.1	(42)
Rat, plantar/gastrocnemius incision	6.54 ± 0.12	(43)
Human, muscle	4.5	(44)
Minipig, intervertebral disc degeneration	5.7	(45)
Rat, chronic sciatic nerve constriction	6.91	(46)
Rat, intraperitoneal acetic acid injection	4.52 (5 min), 6.97 (15 min)	(46)
Mouse, colitis	6.71 ± 0.09	(47)
Mouse, colitis	6.36 ± 0.05	(48)

**Cancer**		

Rat, implanted tumors	6.82	(49)
Human, malignant tumors, inflamed tissues	5.44	(50)
Human, malignant tumor	5.7	(51)
Human, astrocytoma	5.85	(52)
Human, melanoma	6.4	(53)
Mouse, mammary carcinoma	5.8	(54)
Nude mouse, implanted tumors	6.65	(55)
Mouse, implanted tumor	6.66	(56)
Rat, mouse, implanted tumors	6.3	(57)
Mouse, implanted tumors	6.0	(58)
Mouse, implanted subcutaneous lymphoma	6.0	(59)

**Arthritis**		

Human, osteoarthritis, joint injury, synovial fluid	6.5	(60)
Human, rheumatoid arthritis synovial fluid	7.08	(61)
Human, rheumatoid arthritis synovial fluid	6.0	(62)
Human, rheumatoid arthritis synovial fluid	6.4	(63)
Human, rheumatoid arthritis	6.84	(64)
Human, arthritis	6.60	(65)
Human, arthritis, synovial fluid	6.2	(66)
Human, rheumatoid-/osteoarthritis synovial fluid	6.85	(67)
Human, rheumatoid arthritis synovial fluid	7.03	(68)
Horse, staph. aureus-induced arthritis, synovial fluid	6.2	(69)
Rat, BSA-induced arthritis	5.66	(70)

## Protonation state of ligands

**Figure 1:**
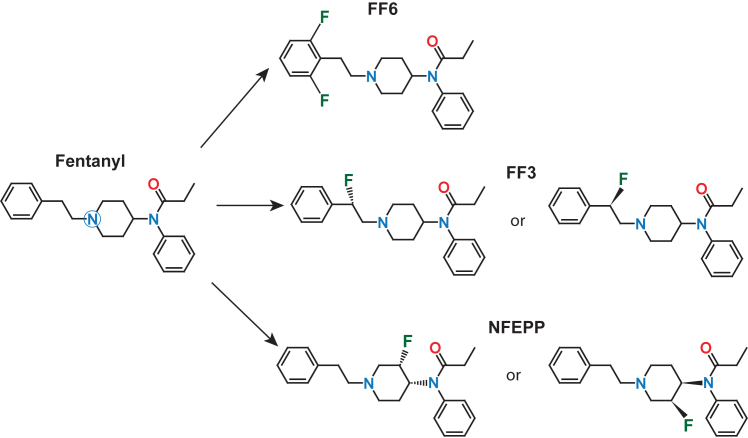
Chemical structures of fentanyl, N-{1-[2-(2,6-difluorphenyl)ethyl]piperidine-4-yl} N-phenylpropionamide (FF6), (±)-*N*-[1-(2-fluoro-2-phenylethyl)piperidine-4-yl]-*N*-phenyl propionamide (FF3), and (±)-*N*-(3-fluoro-1-phenethylpiperidine-4-yl)-*N*-phenyl propionamide (NFEPP). The blue circle highlights the tertiary nitrogen atom subjected to pH-dependent protonation in whose vicinity electrons may be withdrawn to reduce the pKa value. The respective isomers of FF3 and NFEPP are shown. Sites of fluorination are indicated as F. Because FF3 and FF6 elicited some adverse side effects, they were not pursued further (adapted from (114)).

Several previous studies have demonstrated that the function of different GPCRs is pH dependent (19,21,71,72,73,74,75,76,77,78). In MORs, it was shown that ionic interactions between a protonable tertiary amine in the ligand and the amino acid residue D147^3.32^ are particularly important for effective binding and receptor activation (71,79). Within the binding pocket, the protonated amine forms a salt bridge with the negatively charged carboxylate of D147^3.32^ (80,81,82,83). The protonation state of the tertiary amine is dependent on the electron density at the nitrogen atom, which can be influenced by neighboring electronegative moieties such as halogen atoms or other groups (e.g., cyclopropanyl) (84,85,86,87) (Fig. 1). Corresponding observations were made with different GPCRs and ligands (88,89). In addition, the protonation state of H297^6.52^ plays an important role (71,82,90,91). It appears that ligands containing a hydroxyl group (e.g., the peptide DAMGO or the morphinan derivative naloxone) can modulate MOR signaling by forming hydrogen bonds with protonated H297^6.52^ (92). Besides orthosteric binding, possible allosteric interactions with the receptor following pH-dependent absorption of the ligand into the cell membrane also have to be considered (93).

## Protonation state of receptors

Not only ligands but also receptors undergo changes in inflamed microenvironments. There are substantial in silico and in vitro data suggesting that both the structure and function of opioid receptors differ between healthy and inflamed/acidic conditions: 1) computational modeling studies of MORs indicate that the pH-dependent protonation state of functionally important aspartic acid and histidine residues impacts ligand binding rates and hydrogen bond networks that extend throughout the receptor, thereby influencing the intracellular G-protein-coupling dynamics (91,94). 2) Further (in vitro) studies have suggested that H297^6.52^ is critically involved in MOR ligand binding (71,80,81,95,96), its protonation enables hydrogen bonds with specific ligands, and MOR binding and signaling induced by these ligands are pH sensitive (92). In other GPCRs, H^6.52^ was also shown to be important, although its protonation state was not explicitly examined (88,97,98). 3) Additional amino acid residues whose protonation could change at low pH include D^2.50^, E^3.28^, and E^6.30^ (83,99,100). 4) It appears that such pH-dependent changes affect not only the structure/conformation of the receptor itself but also downstream mechanisms of G-protein coupling, arrestin binding, and membrane ion currents (7,101).

## A novel prototype ligand

A novel prototype developed along these lines is (±)-N-(3-fluoro-1-phenethylpiperidine-4-yl)-N-phenylpropionamide (NFEPP) (28) (Fig. 1). At the outset, it was hypothesized that an agonist designed to selectively activate MOR at low pH will not elicit side effects typically mediated by central or intestinal MORs exposed to physiological microenvironments. In computational simulations, structural data on the MOR and its ligand fentanyl were integrated. Because fentanyl’s acid dissociation constant (pKa) is above 7.4 (Table 2), it is protonated and can activate MORs in both normal (pH 7.4) and inflamed (pH 5–7) milieus. It was then hypothesized that a ligand with a pKa between 6 and 7 should be protonated and able to activate MORs exclusively in inflamed tissue. Computational modeling studies simulated the replacement of hydrogens by fluorine in the vicinity of the tertiary amine of the ligand. The fluorine atom was chosen because it is electronegative and decreases pKa and because it would not significantly change the overall size or geometry (and hence the pharmacokinetics) of the derivative compared to the parent compound (fentanyl) (84). In addition, the binding energies of protonated and deprotonated ligands were calculated. The strong interaction between protonated fentanyl and D147^3.32^ in MORs was lost without protonation. The most promising candidate regarding both quantum-chemically estimated pKa values and binding energies was identified. Accordingly, NFEPP was synthesized (Fig. 1). The chemical alteration increased the overall positive charge of its amine (due to the electron-withdrawing effect that fluorine has on neighboring atoms), decreased its pKa to 6.8, and restricted its protonation and binding/activation of MORs to environments with low pH (i.e., high proton concentrations as in injured/inflamed tissue) (28,84,87,94,101).

**Table 2:** pKa values of common opioid ligands

Opioid ligand	pKa
Alfentanil	7.82^a^
Buprenorphine	8.31
Butorphanol	8.19
Codeine	8.21
Fentanyl	8.43
Heroin	7.95
Levorphanol	9.58
Meperidine	8.59
Methadone	8.94
Morphine	8.21
Nalbuphine	8.71
Naloxone	7.94
Oxycodone	8.53
Oxymorphone	8.17
Pentazocine	8.88
Remifentanil	7.51^b^
Sufentanil	7.85
Tramadol	9.41

All values (except a and b) are experimentally measured values from (126).

a Calculated values from ChEMBL, no experimental values available.

b Calculated values from DrugBank, no experimental values available.

Meanwhile, this novel concept has been widely appreciated by other groups (3,4,6,10,82,83,102,103,104,105,106,107,108,109,110,111,112,113) and has been extended to other ligands (e.g., further derivatives of fentanyl or morphine) and algorithms (84,85,86,89,114) (Fig. 1).

By now, multiple in vitro and in vivo investigations performed in our own and external independent laboratories have confirmed that NFEPP indeed blocked sensory neuron excitability and produced efficient analgesia in many rat and mouse models of painful conditions such as arthritis, cancer, nerve injury (neuropathy), bowel inflammation (colitis), abdominal wall inflammation (peritonitis), surgery, and wounds. Initial translational studies confirmed such findings in human sensory neurons (48). Importantly, analgesic doses of NFEPP did not elicit any of the typical adverse effects of conventional opioids (cardio-respiratory arrest, addiction potential, constipation, sedation, locomotor disturbance, tolerance) (28,30,46,47,48,84,114,115,116).

## Implications for drug design

The standard drug development model begins with target selection (e.g., a specific receptor expressed in a disease-relevant tissue) and moves onto high-throughput screens, generation of druggable lead compounds, validation of these compounds in preclinical disease models, and toxicology/safety assessments (117). Drug design strategies employ a range of medicinal chemistry methods to study structure-activity relationships. Both pharmacodynamics (e.g., potency, affinity, efficacy, selectivity) and pharmacokinetics (absorption, distribution, metabolism, excretion, toxicity) are important parameters. Molecular docking, structure-based virtual screening, and molecular dynamics are among the most frequently used strategies in the analysis of molecular recognition events such as binding energetics, molecular interactions, and conformational changes (118).

However, to date, the above described differences between diseased and normal tissues have not been considered. Indeed, these recent findings indicate that traditional drug design is limited by its focus on ligand binding to ubiquitously expressed CPCRs under normal (physiological) conditions (pH 7.4) (80,81,96,119,120,121,122,123,124). Instead, future concepts should take into account that specific microenvironments (e.g., low pH) in different compartments of the body determine the respective ligand-receptor interactions and the resulting desired or undesired effects (94,111,125). In particular, a multi-objective optimal affinity approach was proposed that aims at maximizing the binding affinity to the receptor in injured tissues (at low pH) while minimizing it in healthy environments (at normal pH) (86). In the future, such approaches may include artificial intelligence methods. Opioids are a prominent example: because all available conventional opioid ligands have pKa values above 7.4 (physiological pH) (84) (Table 2), these compounds are protonated at both low and normal pH values. Thus, they can nonselectively bind and activate opioid receptors in both injured/inflamed and healthy (brain, intestinal wall) tissues and simultaneously produce pain inhibition and the above described detrimental side effects. The pH discrepancy between healthy and inflamed tissue provides an opportunity for the design of novel ligands that bind selectively under low pH conditions. Thereby, detrimental side effects mediated by opioid receptors in healthy tissues (brain, intestinal wall) can be avoided. In addition, studies indicate that the pKa of optimal ligands should be close to the local pH of a particular disease state (84,114), calculated pKA values often differ from measured ones (84,126), future opioid drugs should not contain protonable hydroxyl groups (92), pH-sensitive compounds exhibit their maximum efficacy at peak inflammation (48), and local protons (pH) play a more important role than other components of inflammation (e.g., oxygen radicals) (101).

## pH-sensitive ligands in comparison to conventional drugs

As outlined above, pH-dependent GPCR ligands can selectively activate disease-relevant receptors (pathological) while keeping nonrelevant (physiological) receptors (in healthy tissues) untouched. In the case of opioids, the cited in vivo studies have already demonstrated that this concept can give rise to effective analgesics without adverse actions. There is no doubt that novel compounds will only be attractive if they are able to produce desired effects (analgesia) comparable in potency to other available drugs. While the cited animal studies have already demonstrated this, only clinical studies in human patients will finally answer this quest. In the pain medication market, novel pH-dependent opioids will have to compete with ion channel blockers, nonsteroidal anti-inflammatory drugs (NSAIDs), or G-protein-biased agonists (110). So far, studies suggest that pH-dependent opioids will have the following advantages: 1) most painful syndromes (e.g., surgery, arthritis, neuropathy, colitis, cancer) are driven by peripheral sensory neurons (18,127,128). Opioid receptors in these neurons are upregulated, and they can synergistically modulate multiple signaling components (cAMP, Ca^2+^, K^+^, Na^+^, ASIC, HCN, and TRP channels) that decrease electrical excitability (4,5,6,8). This implies a much wider range of efficacy than selective blockers of individual ion channels (e.g., Na_v_1.7, Na_v_1.8, TRPV1). In addition, these ion channels are ubiquitously expressed in many organs (e.g., heart, brain, cerebellum, spinal cord, sympathetic nerves) (particularly under pathological conditions), and therefore, such drugs are bound to produce adverse side effects (e.g., arrhythmia, confusion, ataxia, temperature dysregulation) (129,130,131). 2) Less severe pain states are commonly treated with NSAIDs, but over-the-counter availability and self-medication have also led to frequent abuse and toxicity. All available NSAIDs are associated with detrimental side effects such as bleeding, gastrointestinal ulcer, heart attack, or stroke (132,133). 3) G-protein-biased opioid agonists have been shown to elicit respiratory depression, addiction potential, and constipation (134,135). 4) pH-dependent opioids do not elicit typical adverse effects of conventional opioids.

## Conclusion and outlook

In conclusion, the above described studies strongly suggest distinct ligand-GPCR interactions in healthy versus injured/inflamed milieus. Apparently, local proton concentrations play a decisive role. The structure and function of not only ligands but also receptors clearly differ between those conditions. As demonstrated by, e.g., hydrogen-bonding networks, the geometric configurations (conformations) of opioid receptors localized on peripheral sensory neurons innervating injured/inflamed tissues are not identical to the (normal) ones described so far. Indeed, it appears that peripheral opioid receptors are not the same as those in the central nervous system (e.g., brain). For opioid ligands, the protonation state of tertiary amines is crucial. pH-dependent ligands might constitute a key advance in the fight against the deteriorating opioid epidemic.

Apart from GPCRs that are directly activated by protons (19,20), the pH-dependent activation of MORs by cognate ligands is the first example in the GPCR family. Future drug design should take these aspects into account and depart from traditional concepts based solely on ligand-GPCR interactions within normal (pH 7.4) microenvironments. Such approaches could also include artificial intelligence methods. Given the high degree of homology between GPCRs, these considerations should also apply to other signaling pathways (e.g., from receptor to nucleus or from endosomes), other diseases involving GPCRs (e.g., cancer, arthritis, high blood pressure, addiction, depression, Alzheimer, Parkinson, virus-induced inflammation, atherosclerosis), or even nonhuman GPCRs in deranged environments (e.g., in animals or plants exposed to ocean acidification).

## Author contributions

Writing and editing, C.S.
